# Comparative Morphometry of the Sacrum and Its Clinical Implications: A Retrospective Study of Osteometry in Dry Bones and CT Scan Images in Patients Presenting With Lumbosacral Pathologies

**DOI:** 10.7759/cureus.22306

**Published:** 2022-02-16

**Authors:** Suranjana Banik, Sudipta Mohakud, Sanjukta Sahoo, Prabhas R Tripathy, Simran Sidhu, Manisha R Gaikwad

**Affiliations:** 1 Anatomy, All India Institute of Medical Sciences, Bhubaneswar, Bhubaneswar, IND; 2 Radiology, All India Institute of Medical Sciences, Bhubaneswar, Bhubaneswar, IND; 3 Radiodiagnosis, All India Institute of Medical Sciences, Bhubaneswar, Bhubaneswar, IND

**Keywords:** ct scan, spine, internal fixation, pedicle screw, sacrum

## Abstract

Background

Morphometric measurement of the sacrum is crucial due to its active involvement in the instrumentation for lumbar pathologies. From screw placement to stabilization procedures for the spine, the sacrum remains a site of surgical importance. Thus, the purpose of this study was to generate baseline data by comparing two techniques, namely, osteometry in dry bones and CT scan imaging.

Methodology

In this study, 30 dry, fully ossified, disarticulated sacra were studied for osteometry, and 60 CT scan reports of patients with lumbar pathologies were retrospectively evaluated. In both cases, similar parameters were measured. The mean values were determined, the two methods were compared, and statistical analysis was performed.

Results

Among the 30 dry bone samples, 33.3% (10 out of 30) were males, while 55% of the CT scan group were males. Correlation between the different measurements in the CT scan group suggested that the vertebral body maximum width of S1 had a significant positive correlation with the vertebral body height of S1, sacral height, sacral breadth, transverse diameter of auricular surface, and vertical diameter of auricular surface. Statistically significant higher values (P < 0.001) were observed for the vertebral body mid diameter of S1, vertebral body height of S1, pedicle width, and pedicle depth measurements in the dry bone group compared to the CT scan group.

Conclusions

The efficiency of anaesthetic blocks can be increased if the parameters are evaluated beforehand. Moreover, sexual dimorphism of the bone can account for the varied results of the parameters, indicating the necessity to conduct gender-based studies in a wider population.

## Introduction

Around 400-250 BC, Hippocrates in his collection termed “On the Articulations” first mentioned the bone called the sacrum. The fusion of five sacral vertebrae forms the large triangular bone sacrum. It forms the posterosuperior wall of the pelvic cavity and is sandwiched between the two hip bones. Its caudal end and superior wide base articulate with the coccyx and the fifth lumbar vertebrae at the lumbosacral angle, respectively. Between the base and apex, there are dorsal, pelvic, and lateral surfaces of the sacrum and a sacral canal. The dorsal surface is convex while the pelvic surface is concave. This bone forms a link between the spine and the iliac bones and thus plays an important role in the stability of the hip. Also known as “hieron ostoun,” with hieron meaning “temple” in Greek, the sacrum was considered to be holy because it is the sanctuary of the genitalia [1]. The sacrum is a highly complex bone, and variable anatomy is its characteristic, as documented over the years. Knowledge of its anatomical variation as well as morphometry is essential in the investigation of the diseases of the sacral region, physical examination, as well as operation techniques during surgeries.

Although pelvic fixation is an important treatment modality in adult spinal deformity, it is a challenging procedure that can potentially increase surgical morbidity. Iliosacral screw placement is percutaneously performed and is equal or superior to other techniques of internal fixation concerning safety [2]. Iliosacral screws employed for the stabilization of pelvic ring injuries use a corridor of bone through the ilium, sacroiliac joint, sacral ala, and into the sacral promontory [3]. For this, however, variations of the upper sacral anatomy according to gender and patient morphologic characteristics need to be elaborated for safer placement of the Iliosacral screw in the prone position without dissection of the Iliosacral region. This type of minimally invasive surgery can reduce the risk of complications such as surgical site infections [2].

The knowledge of anatomical variations of the sacral hiatus can improve the reliability of caudal epidural block, as well as strengthen the confidence of the anesthetist and endoscopist and prevent high failure rates and popping through the sacrococcygeal ligament. Hence, determining the anatomical variations of the sacral hiatus is important [4].

This study aims to generate baseline data of various morphometric measurements of the sacrum by osteometry from dry bones and compare it with measurements from a high-end imaging modality such as CT to determine the values critical to orthopedic surgeons, anesthetists, anatomists and forensic experts, implant manufacturers who deal with this bone regularly.

## Materials and methods

Anatomical osteometry

**Figure 1:**
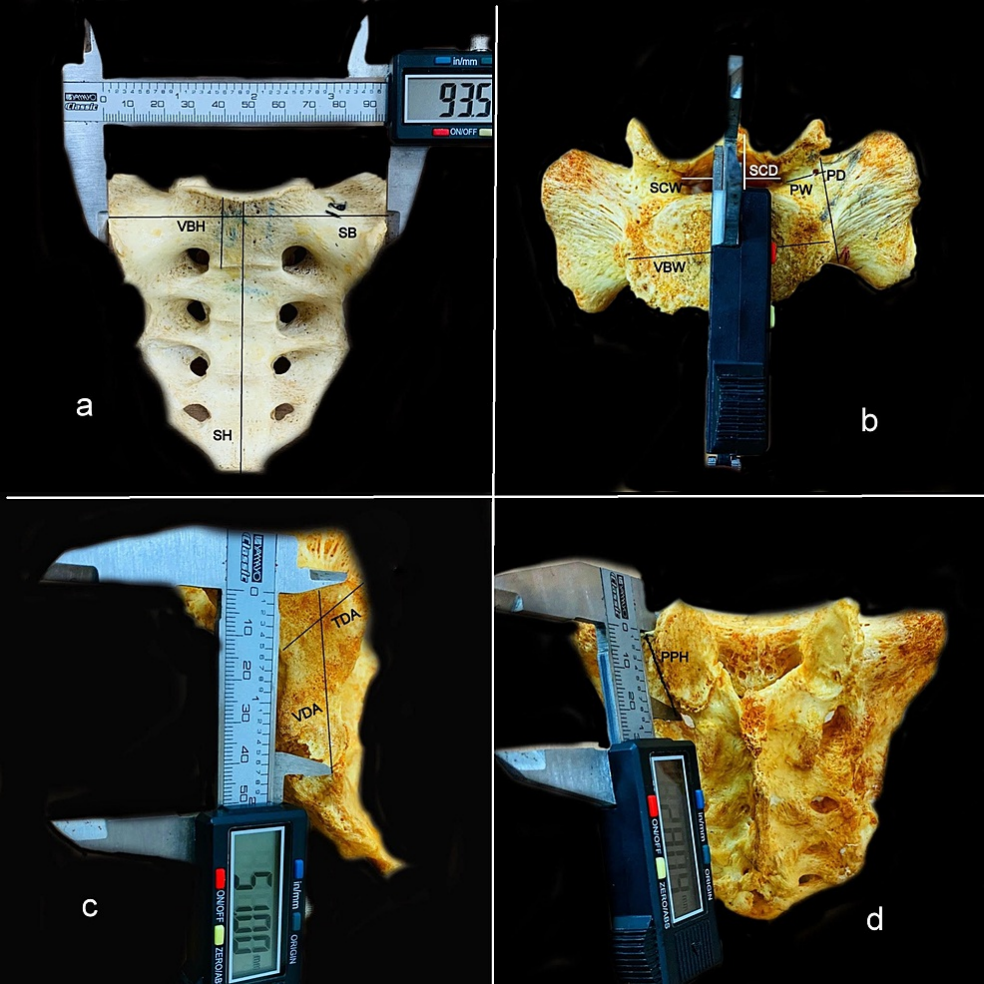
Measurement of sacral parameters in dry bone using a digital Vernier caliper. (a) VBH, SH, and SB. (b) SCW, VBW, SCD, PW, and PD. (c) TDA and VDA. (d) PPH. VBH: vertebral body height of S1; SH: sacral height; SB: sacral breadth; SCW: spinal canal width; VBW: vertebral body maximum width of S1; SCD: spinal canal mid-diameter; PW: pedicle width; PD: pedicle depth; TDA: transverse diameter of auricular surface; VDA: vertical diameter of auricular surface; PPH: posterior pedicle height

We examined 30 dry, fully ossified, disarticulated sacra, which were collected from the Department of Anatomy of All India Institute of Medical Sciences, Bhubaneswar. Broken, deformed, and partially ossified sacra were excluded. The sex and age of the sacrum were not taken into consideration. This was a retrospective study conducted over two months. The S1 segment was especially measured to throw light on the surgical procedures around this region. A digital Vernier caliper with 0.01 mm precision was used (Figure 1) to measure the following diameters: (1) sacral height (SH), the distance from the apex to the base (promontory) of the sacrum; (2) sacral breadth (SB), the widest distance of the alar surfaces; (3) vertebral body maximum width of S1 (VBW), the widest transverse distance of the S1 vertebral body; (4) vertebral body mid-diameter of S1 (VBD), the distance from the anterior to posterior limits of the S1 vertebral body at the midline; (5) vertebral body height of S1 (VBH), the distance between the superior and inferior limits of the S1 vertebral body at the midline; (6) spinal canal width (SCW), the widest transverse distance of the spinal canal; (7) spinal canal mid-diameter (SCD), the distance from the anterior to posterior limits of the spinal canal at the midline; (8) posterior pedicle height (PPH), the distance between the superior limit of the S1 and the superior aspect of the first sacral foramen; (9) pedicle width (PW), the distance from the anterolateral edge of the S1 vertebral body to the lateral edge of the superior facet of S1; (10) pedicle depth (PD), the distance from the anterior to posterior limits of the pedicle at the narrowest point; (11) transverse diameter of auricular surface (TDA), the maximum transverse height of the auricular surface; (12) vertical diameter of auricular surface (VDA), the maximum vertical width of the auricular surface.

All data including mean, minimum, maximum range, and standard deviation were recorded and analyzed using SPSS version 21 (IBM Corp., Armonk, NY, USA). 

CT scan measurements

**Figure 2:**
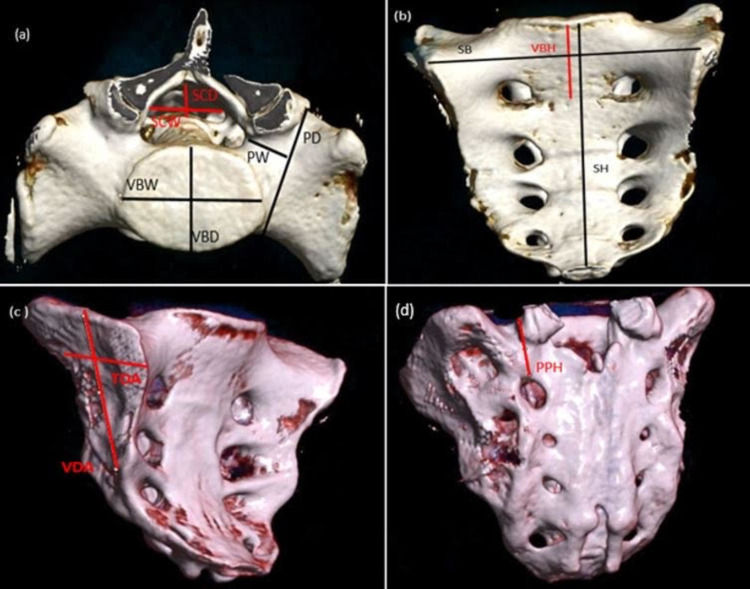
The MDCT shaded surface volume-rendering technique images of the sacrum with its morphometry obtained in a 256-slice CT scanner showing the various measurements. (a) the superior aspect of the sacrum: VBD at the S1 level, VBW, SCD, SCW, PD, and PW. (b) The anterior aspect of the sacrum: VBH at the S1 level, SB, and SH. (c) The lateral aspect of the sacrum: VDA and TDA. (d) The posterior aspect of the sacrum: PPH of the S1 vertebra. MDCT: multidetector computed tomography; VBD: vertebral body mid-diameter; VBW: vertebral body maximum width; SCD: spinal canal mid-diameter; SCW: spinal canal maximum width; PD: pedicle depth; PW: pedicle width; VBH: vertebral body mid-height; SB: sacral breadth; SH: sacral height; VDA: vertical diameter of the auricular surface; TDA: transverse diameter of the auricular surface; PPH: posterior pedicular height

All 60 patients whose CT scan reports were retrospectively evaluated were subjected to examination on a 256-slice MDCT Siemens scanner. The CT protocol of 3 mm multidetector computed tomography (MDCT) sections in the axial plane at a table speed of 5 mm/second (pitch 0.8, rotation time 0.5 seconds) with 120 kV and 147 mAs was used. A plain/non-contrast scan of the pelvis was performed for patients fitting the inclusion criteria with no history of fractures in the pelvic girdle (Figure 2).

Two radiologists assessed three-dimensional reconstruction images of the sacrum by volume-rendering technique and made measurements in consensus.

Statistical analysis

All data were entered in Microsoft Excel 2007 and further analyzed in SPSS version 27 (IBM Corp., Armonk, NY, USA). Quantitative variables were expressed as mean and standard deviation. The difference in means between the two groups was assessed by independent-sample t-test or Mann-Whitney U test. Correlation between two quantitative variables was determined using Pearson’s or Spearman’s correlation coefficients. The normality of the variables was checked using the Kolmogorov-Smirnov test. A P-value less than 0.05 was considered statistically significant.

## Results

This study compared the bony attributes of the sacrum in dry bones with the measurements obtained from CT scans. A total of 90 sacrum bone samples were included and measured in our study, of which 30 were dry bone (henceforth called the dry bone group) and 60 were CT scans of the sacrum (henceforth called the CT scan group). Among the 30 dry bone samples, 33.3% (10 out of 30) were males, while 55% of the CT scan group were males (p-value = 0.053). The mean age of the study participants in the CT scan group was 48.22 (SD 15.19) years, with a range from 17 to 76 years. A comparison of various measurements in the two groups is shown in Table 1.

**Table 1:** Comparison of different parameters between the CT scan (n = 60) and dry bone (n = 30) groups. SH: sacral height; SB: sacral breadth; VBW: vertebral body maximum width; VBD: vertebral body mid-diameter of S1; VBH: vertebral body height of S1; SCW: spinal canal width; SCD: spinal canal mid-diameter; PPH: posterior pedicle height; PW: pedicle width; PD: pedicle depth; TDA: transverse diameter of auricular surface; VDA: vertical diameter of auricular surface #: Mann-Whitney U test was used to calculate P-values, and for the rest of the variables independent-sample t-test was used.

Measurements	CT scan group (mean ± SD)	Dry bone group (mean ± SD)	t-value	P-value
VBW	46.39 ± 5.43	48.26 ± 5.49	-1.53	0.129
VBD	27.39 ± 3.48	32.17 ± 4.21	-5.71	<0.001
VBH	23.44 ± 2.97	30.03 ± 4.09	-8.66	<0.001
SH	96.08 ± 10.81	99.59 ± 15.09	-1.26	0.209
SB	103.29 ± 5.43	99.85 ± 9.42	2.20	0.030
SCW	27.85 ± 4.73	28.00 ± 3.45	-0.15	0.817^#^
SCD	12.47 ± 2.41	20.42 ± 3.13	-13.28	<0.001^#^
PPH	12.99 ± 2.30	22.60 ± 4.23	-13.95	<0.001
PW	7.57 ± 1.59	18.35 ± 2.35	-25.62	<0.001
PD	21.93 ± 5.16	31.79 ± 4.77	-8.74	<0.001^#^
TDA	23.08 ± 4.99	37.03 ± 10.07	-8.80	<0.001
VDA	55.53 ± 6.90	54.73 ± 5.91	0.54	0.560^#^

Apart from the SH and TDA, we observed a higher value for dry bones compared to CT scan measurements. Statistically significant higher values were observed for VBD, VBH, SB, SCD, PPH, PW, and PD measurements in the dry bone group compared to the CT scan group.

Gender differences in the CT scan group suggested a significant difference in higher values for VBW, VBD, VBH, PPH, TDA, and VDA measurements in the CT scan group. In the dry bone group except for the SH (p-value = 0.002), we did not find a statistically significant difference for any other sacral measurements, as shown in Table 2.

**Table 2:** Gender-wise comparison of parameters in the CT scan (n = 60) and dry bone (n = 30) groups. SH: sacral height; SB: sacral breadth; VBW: vertebral body maximum width; VBD: vertebral body mid-diameter of S1; VBH: vertebral body height of S1; SCW: spinal canal width; SCD: spinal canal mid-diameter; PPH: posterior pedicle height; PW: pedicle width; PD: pedicle depth; TDA: transverse diameter of auricular surface; VDA: vertical diameter of auricular surface #: Mann-Whitney U test was used to calculate P-value, and for the rest of the variables independent-sample t-test was used.

Variables	CT scan group (mean ± SD)			Dry bone group (mean ± SD)		
	Male (n = 34)	Female (n = 26)	P-value	Male (n = 10)	Female (n = 20)	P-value
VBW	48.1 ± 5.5	44.1 ± 4.5	0.004	48.1 ± 7.2	48.3 ± 4.5	0.941
VBD	28.3 ± 3.4	26.1 ± 3.1	0.018	32.1 ± 3.3	32.2 ± 4.6	0.951
VBH	24.6 ± 2.5	21.8 ± 2.7	<0.001	29.5 ± 3.8	30.2 ± 4.3	0.704
SH	98.0 ± 10.2	93.5 ± 11.1	0.115	111.0 ± 15.1	93.8 ± 11.6	0.002
SB	103.0 ± 6.0	103.5 ± 4.6	0.727	99.3 ± 11.2	100.0 ± 8.6	0.746^#^
SCW	27.5 ± 5.2	28.3 ± 4.0	0.765^#^	26.3 ± 3.1	28.8 ± 3.3	0.070
SCD	12.5 ± 2.6	12.3 ± 2.0	0.799	19.5 ± 2.7	20.8 ± 3.3	0.502^#^
PPH	13.5 ± 2.3	12.2 ± 2.0	0.021	21.2 ± 3.3	23.2 ± 4.5	0.210
PW	7.6 ± 1.5	7.5 ± 1.6	0.788^#^	19.0 ± 2.1	17.9 ± 2.4	0.233
PD	22.8 ± 5.4	20.7 ± 4.6	0.179^#^	29.8 ± 4.7	32.7 ± 4.6	0.114
TDA	24.7 ± 5.2	20.9 ± 3.6	0.005^#^	35.0 ± 8.4	38.0 ± 10.8	0.460
VDA	58.2 ± 7.1	51.9 ± 4.6	<0.001	55.5 ± 6.7	54.3 ± 5.5	0.779^#^

Correlation between the different measurements in the CT scan group suggested that VBW had a significant positive correlation with VBH, SH, SB, TDA, and VDA. VBH showed a high positive correlation with pedicle and articular surface parameters of the sacral bone, as depicted in Table 3.

**Table 3:** Correlation between different parameters in the CT scan group (N = 60). SH: sacral height; SB: sacral breadth; VBW: vertebral body maximum width; VBD: vertebral body mid-diameter of S1; VBH: vertebral body height of S1; SCW: spinal canal width; SCD: spinal canal mid-diameter; PPH: posterior pedicle height; PW: pedicle width; PD: pedicle depth; TDA: transverse diameter of auricular surface; VDA: vertical diameter of auricular surface Values in each cell show the correlation coefficients (r). *P-value < 0.05 and **P-value < 0.01.

	VBW	VBD	VBH	SH	SB	SCW	SCD	PPH	PW	PD	TDA	VDA
VBW	1											
VBD	0.2	1										
VBH	0.3^*^	0.1	1									
SH	0.3^*^	-0.1	0.2	1								
SB	0.3^*^	-0.1	0.1	0.2	1							
SCW	0.1	-0.2	-0.1	0	0.3^**^	1						
SCD	0.1	0	-0.1	0.3^*^	0.1	0.3^*^	1					
PPH	0.2	0	0.4^**^	0.1	-0.1	-0.2	0	1				
PW	0.2	-0.2	0.2	0	0.1	-0.1	-0.1	0.5^**^	1			
PD	0.2	-0.2	0.3^**^	-0.1	0	0	-0.2	0.5^**^	0.4^**^	1		
TDA	0.3^**^	0.2	0.4^**^	0	0.1	0	-0.2	0.4^**^	0.3^**^	0.2	1	
VDA	0.4^**^	0	0.4^**^	0.3^*^	0.2	0.2	0.2	0.1	-0.1	0.2	0.4^**^	1

Similarly, in the dry bone group, significant positive correlations were observed for VBD with SCW, SCD, and all the pedicle parameters of the sacral bone. SCW also showed a highly significant positive correlation with SCD, PPH, PD, and TDA. Details are presented in Table 4.

**Table 4:** Correlation between different parameters in the dry bone group (N = 30). SH: sacral height; SB: sacral breadth; VBW: vertebral body maximum width; VBD: vertebral body mid-diameter of S1; VBH: vertebral body height of S1; SCW: spinal canal width; SCD: spinal canal mid-diameter; PPH: posterior pedicle height; PW: pedicle width; PD: pedicle depth; TDA: transverse diameter of auricular surface; VDA: vertical diameter of auricular surface Values in each cell show the correlation coefficients (r). *P-value <0.05 and **P-value <0.01.

	VBW	VBD	VBH	SH	SB	SCW	SCD	PPH	PW	PD	TDA	VDA
VBW	1											
VBD	0.5^**^	1										
VBH	0.5^**^	0.8^**^	1									
SH	0.3	-0.2	-0.1	1								
SB	0.3	0.4^*^	0.2	0.4	1							
SCW	0.5^**^	0.5^**^	0.5^**^	-0.2	0.3	1						
SCD	0.4^*^	0.7^**^	0.7^**^	-0.1	0.4^*^	0.5^**^	1					
PPH	0.1	0.6^**^	0.6^**^	-0.4^*^	0.2	0.5^**^	0.4^*^	1				
PW	0.6^**^	0.5^**^	0.3	0.4	0.3	0.3	0.4^*^	0.1	1			
PD	0.2	0.7^**^	0.6^**^	-0.4^*^	0.3	0.7^**^	0.4^*^	0.8^**^	0.3	1		
TDA	0.4^*^	0.4^*^	0.3	-0.1	0.3	0.7^**^	0.2	0.4^*^	0.5^**^	0.7^**^	1	
VDA	0.3	0.3	0.4^*^	0.1	0.2	0.3	0.4^*^	0.2	0.5^**^	0.4^*^	0.5^**^	1

**Figure 3:**
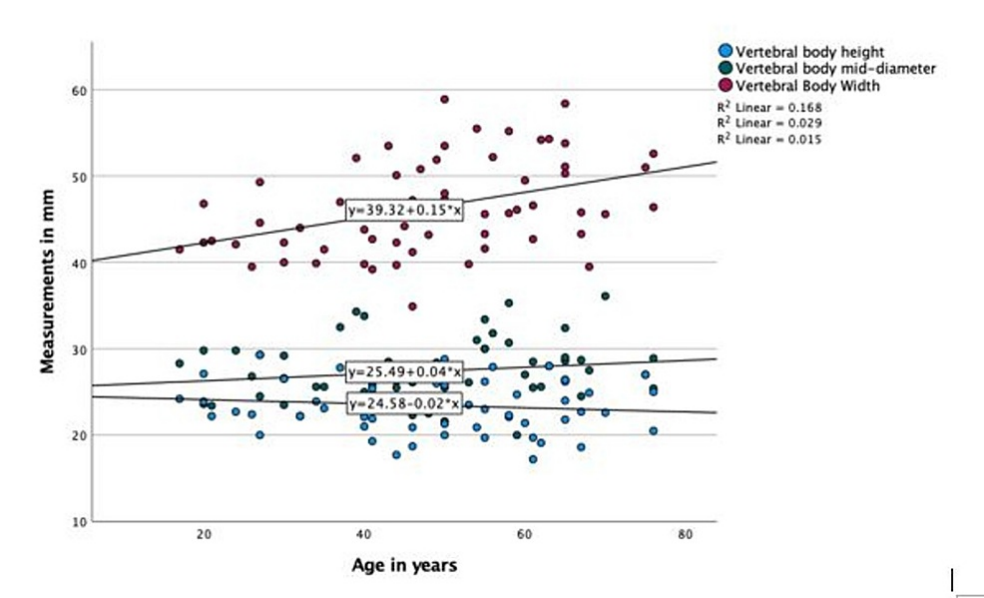
Scatter plot showing the correlation of age with sacral vertebral measurements in the CT scan group.

A positive linear correlation was found between age and vertebral sacral body height, while no correlation and slightly negative correlations were observed for vertebral body diameter and width, respectively (Figure 3).

**Figure 4:**
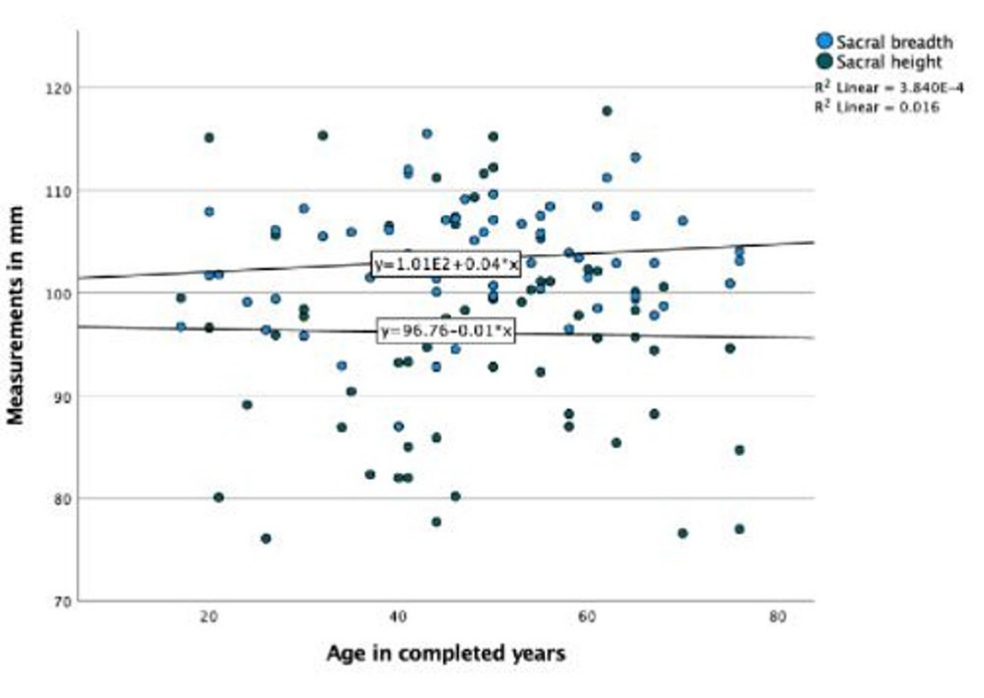
Scatter plot showing the correlation of age with sacral height and breadth in the CT scan group.

The age of the participants showed a non-significant and mild correlation with SH and SB (Figure 4).

**Figure 5:**
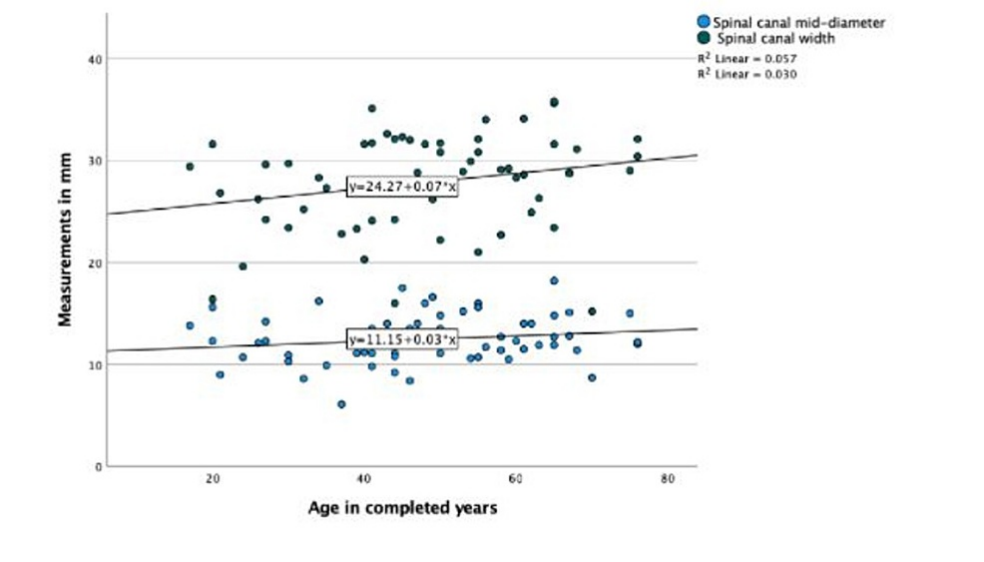
Scatter plot showing the correlation of age with spinal canal measurements in the CT scan group.

 Spinal canal parameters did not have a significant correlation with age (Figure 5).

**Figure 6:**
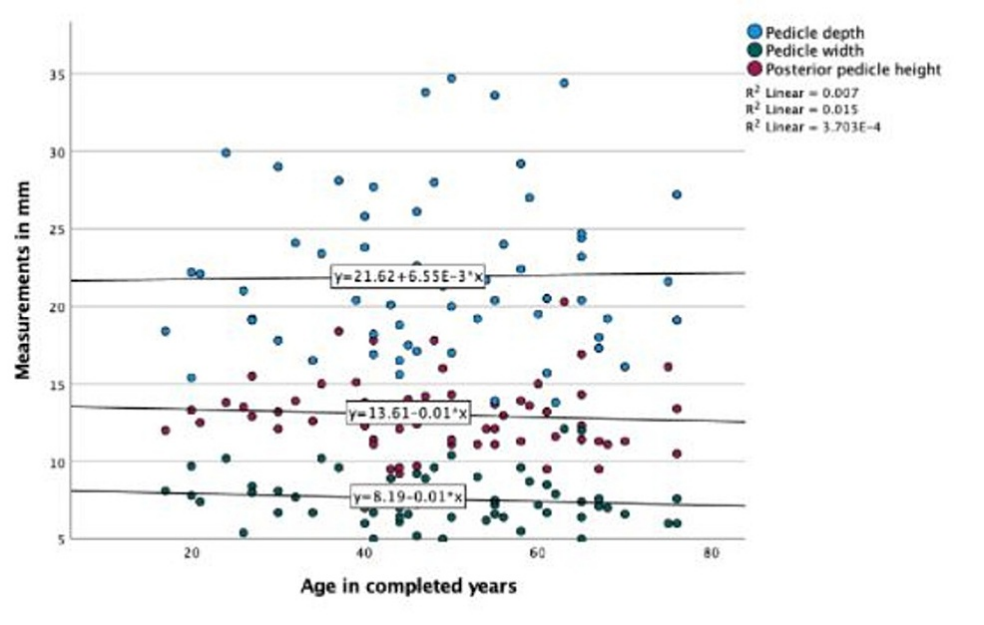
Scatter plot showing the correlation of age with sacral pedicle measurements in the CT scan group.

**Figure 7:**
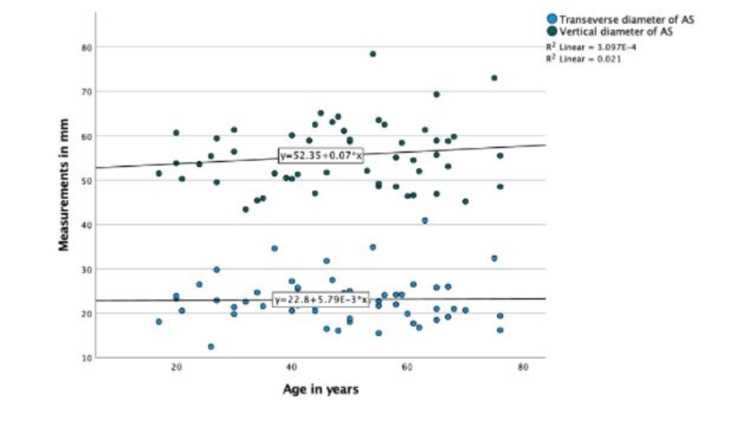
Scatter plot showing the correlation of age with articular surface measurements in the CT scan group.

No correlations between age with pedicle parameters and articular parameters were seen in the CT scan group (P-value > 0.05) (Figures 6, 7).

## Discussion

The S1 vertebra remains a crucial point for spinal instrumentation because the pedicle screw fixation should be in the anteromedial direction into the S1 vertebra [5]. A CT scan study showed that 17% of spina bifida occulta cases impacted the S1 vertebra [6]. Neurovascular structures are at risk with the placement of S1 pedicle screws. These structures include the L5 nerve root crossing over the sacral ala and the S1 nerve root passing through the S1 foramen. It has been observed that the average distance of intraoperative screw placement from the S1 foramen is 3 mm while the screws appear to enter the S1 foramen. This risk can be minimized with the prior assessment [7]. The internal iliac vein and the lumbosacral nerve trunk are mostly at risk for injury by 30 and 45 degrees laterally directed screws. The sigmoid colon is protected by its mesentery, though close to the S2 screw. S1 pedicle screws are least likely to injure the neurovascular bundle. Thus, prior to instrumentation, outcome analysis is required [8]. For stability and reduced rate of failures of instrumentation in scoliotic patients, when extension of the fixation device to the sacrum is indicated, the iliosacral screw placement is indicated, warranting the thorough knowledge of the S1 vertebra morphometry [9].

Basaloglu et al. showed that there was no significant gender difference for SH, but SB had a statistically significant difference (p < 0.05). In their study, the mean SCW in females was 2.97 cm, and in males it was 3.10 cm, which was statistically significant (p < 0.05). The SCD was 1.55 cm in females and 1.46 cm in males, with no significant difference [10].

In our study, gender differences in CT scans showed significantly different higher values for VBW, VBD, VBH, PPH, TDA, and VDA. Except for SH (P-value = 0.002), a statistically significant difference in any other sacral measurements was not observed in the dry bone group. Significant positive correlations were observed for VBD with SCW and SCD in the dry bone group.

In the present study, the mean SH and SB in both genders were estimated to be 9.8 cm in males and 9.35 cm in females and 10.30 cm in males and 10.35 cm in females, respectively, in the CT scan group, whereas in the dry bone group the values were 11.10 cm in males and 9.38 cm in females and 9.93 cm in males and 10 cm in females, respectively. However, few studies have calculated SH and SB in both genders [11,12].

In a study by Xu et al., the mean VBH was 2.89 cm in males and 2.77 cm in females [13]. In the present study, it was 2.46 cm in males and 2.18 cm in females in the CT scan group, while it was 2.95 cm in males and 3.02 cm in females in the dry bone group.

The mean PW was 1.09 cm in males and 1.04 cm in females. In the study by Basaloglu et al., the value in males and females was 1.42 cm, ranging from 1.0 cm and 1.8 cm [10]. In this study, the mean PW in the CT scan group was 0.76 cm in males and 0.75 cm in females, whereas, in the dry bone group, it was 1.9 cm in males and 1.79 cm in females. This data is crucial for screw fixation.

The value of the mean PD, as reported by Ebraheim et al., was 2.78 cm [9]. In this study, PD was 2.28 cm in males and 2.07 cm in females in the CT scan group, whereas in the dry bone group it was 2.98 cm in males and 3.27 cm in females.

Sacral morphometry has been found to be more accurate on CT scans. Moreover, three-dimensional CT scans have shown that the junction between the pedicle and the vertebral body remains the critical point for instrumentation of the spine [14]. Thus, the osteometry parameters were correlated with CT scan measurements in this study. According to our findings, significant positive correlations were noted between different parameters such as VBW with VBH, SH, SB, TDA, and VDA in CT scans, and in the dry bone group, VBD had positive correlations with PD, PW, SCW, and SCD.

The anatomy and measurements of the S1 pedicle and its spatial relationship with the outer surface of the ilium are necessary for the placement of screws, and the use of a CT scan along with fluoroscopic intraoperative guidance is helpful. Moreover, the effective S1 pedicle height may show variable results when measured by CT and gross osteometry due to the difficulty in measuring the concavity of the superior wall of the S1 anterior foramen by the latter method [9]. During intraoperative procedures, when the patient is prone, a modified inlet or outlet view with 45-degree cephalad or caudal angulation of the X-ray beam can provide a better view [15]. Thus, in planned cases, a comparison of mean values generated by both methods can yield accurate results and guide instrumentation.

In a study by Dubory et al., spinopelvic fixation with iliosacral screws was modeled and assessed on 10 CT scans. The study found that the thickness of the inferior part of the S1 vertebral body increased in male patients (p < 0.001). In this study, the vertebral body width in males and females had no significant difference. However, vertebral body mid-diameter and vertebral body height had significant differences (p < 0.001) in both sexes on both osteometry and CT scans. Apart from conventional radiography, CT axial imaging is the ideal modality for studying the normal anatomy of the sacroiliac joint as well as pathologies. The epiphysial ossification centers of the S1 vertebra, as well as the auricular surface, are best demonstrated on a CT scan [16].

In this study, the transverse and vertical diameters of the auricular surface had significant differences (p < 0.001) in males and females when measured both by CT scan, thus re-establishing the sexual dimorphism explained in other studies [17,18]. Because age, gender, and race can affect the morphometry of the sacrum, different ethnic groups and geographical regions should be investigated. The smaller sample size is a limitation of this study, thus, warranting a larger sample size for cross-evaluation of the findings.

## Conclusions

Different anatomical parameters were measured in this study by osteometry on dry sacra as well as by morphometry on CT scan. There was a significant positive correlation in the values of VBW, VBD, SCW, SCD, PD, and VDA by osteometry and CT scan, highlighting that the mean values generated can be used as reference values during various surgical procedures and instrumentation of the sacral region. Gender variations in different parameters showed that the concept of sexual dimorphism and consecutive changes in parameters should be kept in mind. We believe that if the study can be extended into different ethnic groups, then the parameters can be pooled based on racial variations, yielding more information. Thus, the study can generate sufficient knowledge for percutaneous surgical procedures, for maintaining safe zones during usage of implants, and for reducing peri and postoperative complications.
